# Genetically determined alcohol consumption and cancer risk in Korea

**DOI:** 10.4178/epih.e2023077

**Published:** 2023-08-23

**Authors:** Keum Ji Jung, Ji Woo Baek, Sang Yop Shin, Sun Ha Jee

**Affiliations:** 1 Institute for Health Promotion, Department of Epidemiology and Health Promotion, Graduate School of Public Health, Yonsei University, Seoul, Korea; 2 Korea Medical Institute, Seoul, Korea

**Keywords:** Genetics, Causality, Alcohol drinking, Neoplasms

## Abstract

**OBJECTIVES:**

The purpose of this study was to determine the causal relationship between the genetically determined amount of alcohol consumption and the occurrence of major cancers.

**METHODS:**

The data used in this study were from 129,324 people selected from the Korean Cancer Prevention Study-II, the participants of which visited 18 health examination centers between 2004 and 2013. Cancer incidence was confirmed as of 2020 using data from the National Cancer Center. A genome-wide association study (GWAS) on alcohol consumption was performed using PLINK 2.0, and sex, age, chip type, and principal components were adjusted.

**RESULTS:**

From the GWAS, a genetic risk score for alcohol consumption was calculated and genetically determined alcohol consumption (GDAC) was estimated. GDAC was divided into quintile groups and showed significant causal relationships with rectal cancer and liver cancer, but not with other cancers. For liver cancer, an association was shown in the hepatitis B surface antigen (HBsAg)-negative group, and a particularly strong association was found in the over-60-year-old HBsAg-negative group, in which, compared to the GDAC Q1 group, the Q4 group had a 2.35 times higher risk (95% confidence interval [CI], 1.05 to 5.23), and the Q5 group had a 2.40 times higher risk (95% CI, 1.09 to 5.30).

**CONCLUSIONS:**

The results of this study provided evidence that the amount of alcohol consumed is causally related to the occurrence of rectal cancer and liver cancer in HBsAg-negative individuals. Additional studies should be continued for other cancer types through long-term follow-up.

## GRAPHICAL ABSTRACT

[Fig f3-epih-45-e2023077]

## INTRODUCTION

Alcohol drinking is one of the most common lifestyle habits, along with smoking, and many epidemiological studies have been conducted because alcohol consumption has a negative effect on health [1,2]. Observational studies on drinking alcohol and cancer have shown various results in terms of the relationships, but there is not enough evidence to draw a clear conclusion regarding causality [3,4].

Moreover, since most studies related to alcohol drinking are observational studies, it is difficult to completely control for confounding variables, limiting the ability to interpret the relationship [5]. Gene-based Mendelian randomization (MR) is a recently developed way to solve this problem, making it possible to draw conclusions regarding causal association under the assumption that genes are randomly assigned while avoiding the influence of confounding variables. In particular, since many genetic variants related to drinking have recently been discovered and reported, research on causality has become more possible [6,7]. Research in Asians is needed, since genetic factors related to drinking are significantly different between Asians and Westerners [7]. This study investigated the causality between genetically determined alcohol consumption (GDAC) and cancer incidence in Koreans.

## MATERIALS AND METHODS

The data used in this study are from the Korean Cancer Prevention Study (KCPS)-II biobank and were collected from 160,407 subjects who visited 18 comprehensive examination centers, including 15 centers in Seoul and Gyeonggi Province and 3 centers in other regions, from 2004 to 2013 for examinations [8]. Of these, 129,324 people with complete data on measurements of alcohol consumption and genetic information were selected as study subjects. In this study, GDAC and cancer incidence were investigated as follows.

### Data collection

At baseline, all participants were asked to describe their smoking habits and alcohol consumption based on a standardized questionnaire for core variables. Serum hepatitis B surface antigen (HBsAg) was tested by radioimmunoassay or reverse passive hemagglutination in hospital laboratories. The median follow-up period was 13 years, from January 1, 2007 to December 31, 2020.

### Genotyping procedures and genome-wide association study

In order to estimate the GDAC, all subjects needed genetic test data. For 50% of the subjects in this study, a global screening array (GSA) chip was used for testing [9], while testing for the remaining 50% was conducted using the Korea Biobank Array [10]. Then, based on 1,000 genomes in an identical way, imputation was performed using IMPUTE 5 to build integrated data. IMPUTE 5 is a software program designed for imputing and estimating unobserved and missing genotype data of individuals using known haplotype panels and recombination maps. Principal component analysis (PCA) was performed to identify batch effects that could be included in a 159K-sample dataset consisting of GSA and Korean Biobank Array. The number of markers common to all chips was 94,735, and markers with greater than 5% difference in the minor allele frequency (MAF) between platforms were removed. Basic quality control and linkage disequilibrium pruning analysis with applied plink options “ –geno 0.1 –hwe 1e-3 –maf 0.1 –not-chr –mind 0.1 –indep-pairwise 200 50 0.2” were conducted. Through this filtering process, 26,165 markers were identified, and PCA was performed on them. A program called “flash PCA” was used for PCA with a large sample, and the analysis results did not confirm any chip-to-chip batch effect. After confirming the quality control criteria for GWAS analysis (MAF=0.01, Hardy-Weinberg equilibrium < 10^-6^), 6,804,815 single-nucleotide polymorphisms (SNPs) were analyzed. In order to estimate the effect size of each SNP for the amount of alcohol consumption, which was measured as an independent variable, linear regression including sex, age, chip type, and the principal component was conducted. In this study, genome-wide association study (GWAS) analysis was performed using PLINK 2.0.

#### Genetic risk score calculation of drinking amount and genetically determined alcohol consumption

MR was performed using 2-stage least squares regression, with a genetic risk score (GRS) as an instrumental variable. In the previous GWAS analysis, the significance of SNPs that were significantly related to drinking was selected by setting p < 5 × 10^-8^ through the Bonferroni correction for multiple comparisons. The GRS for alcohol consumption was obtained through equation 1 as follows [11]:

Equation (1): GRS value for k SNPs of the th person (*GRS_i_*)


$$GRS_{i}=\sum_{j=1}^{k}\;Number\;of\;risk\;alleles\;in\;SNP_{j}\times Weight_{j},\;i=1,\;...,\;n,\;j=1,\;...,\;k$$

*n*: total number of subjects, *SNP_j_*: jth SNP, *Weight_j_*: Weight of jth SNP

The genetically determined amount of drinking was obtained by predicting the amount based on the GRS value after linear regression analysis of GRS and the measured drinking amount.

### Cancer occurrence ascertainment

Cancer incidence in study subjects was confirmed as of December 2020 by linking cancer registration data of the National Cancer Center [12,13]. This study included esophagus cancer, head and neck cancer, colorectal cancer, liver cancer, stomach cancer, lung cancer, thyroid cancer, female breast cancer, and alcohol-related cancer. Head and neck cancer included oral cavity cancer, pharynx cancer, and larynx cancer. Alcohol-related cancer included oral cavity cancer, pharynx cancer, larynx cancer, esophagus cancer, liver cancer, colon cancer, rectum cancer, and female breast cancer. Based on the resident population in 2020, the age-standardized incidence rate for each cancer type was calculated.

### Analysis of genetically determined alcohol consumption and risk of cancer

In this study, GDAC was divided into quintiles, and the risks of the Q2, Q3, Q4, and Q5 groups were analyzed, compared to that of the Q1 group. The hazard ratio analysis for each group was performed using a Cox proportional hazard model after controlling age and sex. The genetically attributable fraction (GAF)— that is, the degree to which the genetically determined amount of drinking contributes to the occurrence of cancer—was calculated as shown in equation 2 below.

Equation (2)

For statistical analysis in this study, R version 4.1.2 (R Foundation for Statistical Computing, Vienna, Austria) and SAS version 9.4 (SAS Institute Inc., Cary, NC, USA) were used.


$$GAF=\frac{\left[(P_{0}+(P_{1}\times RR_{1})+(P_{2}\times RR_{2})+(P_{3}\times RR_{3})+(P_{4}\times RR_{4}))-1\right]}{\left[P_{0}+(P_{1}\times RR_{1})+(P_{2}\times RR_{2})+(P_{3}\times RR_{3})+(P_{4}\times RR_{4})\right]}\;=\frac{\left[(P_{0}+\sum_{i=1}^{4}(P_{i}\times RR_{i}))-1\right]}{\left(P_{0}+\sum_{i=1}^{4}(P_{i}\times RR_{i})\right)},\;i=1,\;...,4$$

*P_i_*: *Q_i_* prevalence of the group, *RR_i_*: *Q_I_* group relative risk

### Ethics statement

This study protocol was reviewed and approved by the Institutional Review Board of Severance Hospital (Seoul, Korea) and the informed consent was received (IRB No: 4-2011-0277).

## RESULTS

The average age of the 129,324 study subjects was 41.4 years (41.8 years old for male, 40.7 years old for female), the mean follow-up period was 12.8 years, and the total number of person-years (PY) was 1,653,176. During the follow-up period, the number of cancer occurrences (age-adjusted incidence per 100,000 PY) was 9,487 (817.9) for all cancers, 380 (35.8) for liver cancer, 791 (74.0) for colorectal cancer, 1,140 (101.8) for stomach cancer, and 669 (77.9) for lung cancer.

**Figure 1.**
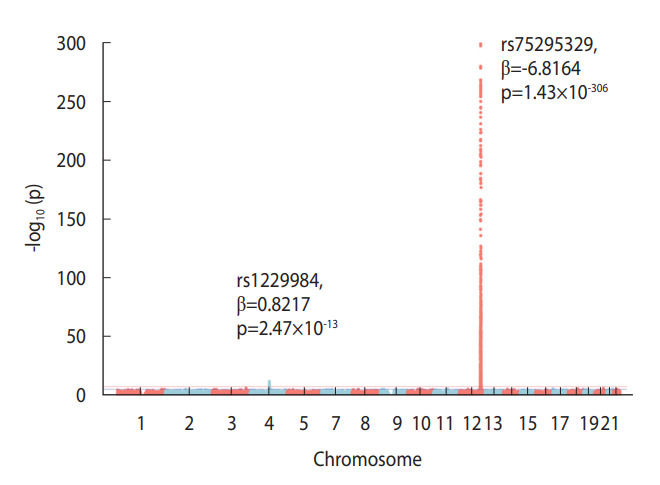
Causal association between genetically determined alcohol consumption and liver cancer risk among subjects aged over 60 with hepatitis B surface antigen negativity. HR, hazard ratio.

The GWAS analysis of the amount of drinking among the study subjects showed strong associations with SNPs related to drinking on chromosomes 4 and 12 (Figure 1). In this study, chromosome 4 contained 511,036 SNPs, and chromosome 12 contained 324,666 SNPs. On the former, 88 SNPs with p < 5 × 10^-8^ were identified, and they were clumped at r^2^=0.01, leaving only 2 SNPs (rs1229984, rs2075633). This limited the number of SNPs to be too small to calculate the GRS. Next, 2,240 SNPs with p< 5× 10^-8^ were identified on the latter, and the top SNP was rs75295329, (p=1.4×10^-306^). When these 2,240 SNPs were clumped at r^2^=0.01, 17 SNPs remained and the GRS of the amount of drinking was calculated. The amount of drinking genetically determined by chromosome 12 demonstrated a distribution between approximately 3 g and 25 g. However, the actual amount of drinking according to the questionnaire showed a distribution from 0 g (nondrinking) to a maximum of 200 g (data not shown).

Table 1 shows the general characteristics according to the GDAC divided into quintiles. The average GDAC increased linearly from 9.3 g/day in Q1 to 21.7 g/day in Q5. Approximately 25,860 subjects were included per group. Age and body mass index in each group demonstrated similar average values per group, although statistically significant differences were observed. However, there was no significant difference in the distribution of sex, smoking experience, and exercise experience among each group. Moreover, the amount of alcohol consumption measured through the questionnaire was the lowest (7.9 g/day) in the Q1 group, and increased by about two times to 14.5 g/day in the Q2 group, and slightly increased after the Q3 group.

Table 2 shows the relationship between all cancer risk and the risks of major cancers according to GDAC. Overall, except for colorectal cancer and liver cancer, there was no difference in cancer risk according to GDAC. For colorectal cancer, a significant association was shown in the Q2, Q4, and Q5 groups compared to the lowest Q1 group. In other words, as the GDAC increased, a slightly increasing relationship was shown (p for trend=0.003). For liver cancer, the risk was 1.49 times higher in the Q4 group compared to the Q1 group, and no significant risk elevation was shown in the other groups (p for trend=0.048). Considering the Bonferroni correction (p_bon_=0.05/10 =0.005), only colorectal cancer was statistically significant (Table 2).

Table 2 shows the results of an in-depth analysis of colorectal cancer, showing the relationship between the risk of colon cancer and rectal cancer according to the 5 groups of GDAC. Taking Q1 as a reference to explore the risks of the other groups, the risk of colon cancer increased by 36% in Q4. In the same comparison, the risk of rectal cancer increased by 47% in Q2, 70% in Q4, and 60% in Q5 (p for trend=0.006).

Table 3 shows the relationship between GDAC and liver cancer according to HBsAg positivity. Overall, in the HBsAg-positive group, GDAC was not associated with liver cancer. However, in the HBsAg-negative group, GDAC displayed a moderate association with liver cancer (p for trend=0.034). When the age was divided into under and over 60, a clear relationship was found, with no relationship at all in those younger than 60 years old, but a 2.35-fold (95% confidence interval [CI], 1.05 to 5.23) risk elevation in Q4 and a 2.40-fold (95% CI, 1.09 to 5.30) risk elevation in Q5 compared to Q1 (p for trend=0.008). The estimated risk of GDAC contributing to liver cancer in the HBsAg-negative group was 41.7% (not shown).

**Figure 2.**
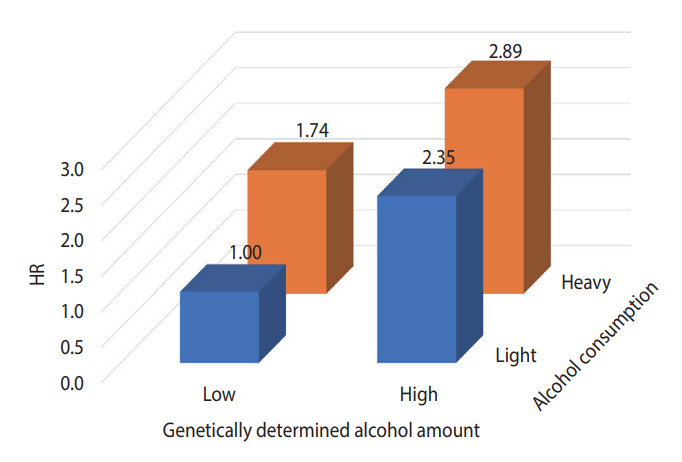
Manhattan plot of alcohol consumption in the Korean Cancer Prevention Study-II Biobank.

Figure 2 presents the results of analyzing the risk of liver cancer in each group after dividing the GDAC into low and high, and dividing the actual amount of drinking into light and heavy, targeting the HBsAg-negative group aged 60 years and older. The risk of liver cancer was 3.1 times higher in the group with high GDAC and heavy drinking than in the group with low GDAC and light drinking.

## DISCUSSION

In this study, the risk of major cancers according to GDAC was analyzed using the KCPS-II Biobank. No evidence was found of a causal relationship between GDAC and all cancers, but a causal relationship was partially shown for rectal cancer and liver cancer.

In the GWAS analysis of this study, genetic variants associated with the amount of drinking measured by the questionnaire demonstrated a very strong association with the *ALDH2* gene on chromosome 12. However, the signal on chromosome 12 reported in this study exists only in East Asia including in Japan, China, and Korea, whereas this phenomenon was not found in Western countries [7]. Instead, genetic variants related to alcohol consumption on chromosomes 2, 3, and 4 have shown strong associations in Westerners [14]. Therefore, it is necessary to conduct additional research related to drinking in Asian countries.

In this study, the age-standardized cancer incidence rates per 100,000 PY calculated in KCPS-II Biobank subjects and the age-standardized cancer incidence rates per 100,000 people reported by the National Cancer Center were compared, and the results were similar except for total cancers and stomach cancer. Specifically, when comparing the age-standardized cancer incidence rates of KCPS-II and the National Cancer Center, those of total cancers were 817.9 per 100,000 PY and 708.6 per 100,000 people, the rates for liver cancers were 35.8 and 38.5, those for colorectal cancers were 74.0 and 70.8, the rates for stomach cancers were 101.8 and 67.5, and those for lung cancers were 77.9 and 73.6, respectively [15]. Kang et al. [15] used Segi’s world standard population targeting citizens aged 0 and older. Therefore, a direct comparison was difficult because our data were cancer incidence data tracked in adults aged 20 years or older. Thus, through the data of the National Statistical Office in 2019, in the population aged 20 years or older, the number of cancers by age at 10-year intervals and the incidence rate were recalculated with the 2019 population. KCPS-II presents the age-adjusted incidence rate calculated through the direct method, using the same 2019 population as the standard population used in the National Statistical Office data. Further research is needed to clarify the difference in stomach cancer in the comparison of the incidence rates of the 2 groups.

HBsAg has been identified as the strongest risk and causative factor for liver cancer [16,17]. In a study by Jee et al. [18] in 2004, the population-attributable risk of HBsAg positivity for liver cancer was 66.7%. However, as the prevalence of HBsAg has been greatly reduced in recent years, it is expected that its populationattributable risk has also substantially decreased [19]. Therefore, it is necessary to study the contribution of other risk factors to liver cancer. For example, alcohol drinking and obesity contribute to the development of liver cancer. In the HBsAg-negative group, which accounted for about 97% of the subjects of this study, the risk of liver cancer escalated as GDAC increased (Table 3). In particular, the risk was 2.40 (95% CI, 1.09 to 5.30) times higher in the Q5 versus Q1 comparison in the population aged 60 years and older. In this study, the attributable risk of GDAC for liver cancer development in those group was 41.7%. However, as shown in Figure 2, even with a GDAC, the risk of liver cancer is lowered if the person does not consume alcohol. This indicates an important public health message. In Korea, the prevalence of HBsAg will decrease through the national HBsAg vaccine project [19], and as the elderly and obese populations increase, research on the effects of drinking or obesity on liver cancer in terms of genetic predisposition should be continued [20].

According to data from Korea Military Manpower Administration medical examinations for conscription from 2003 to 2019 (n =5,355,941), the prevalence of HBsAg in 19-year-old adults decreased from 3.19% to 0.18% [19].

A study was published using UK Biobank data in 2022 to study the relationship between drinking and liver cancer [21]. Hepatocellular carcinoma (HCC) occurred in 201 out of 329,164 UK Biobank subjects, and the measured pure alcohol intake showed a J-shape relationship with the risk of liver cancer, with the lowest risk found at 17.3 g/day. However, in the non-linear MR analysis, the J-shape disappeared and a linear relationship was found. The authors suggested that further studies are needed, as this was the first preliminary study with a small number of HCC cases. Another study, also published in 2022, utilized data from the BioBank of Japan (BBJ), which consisted of approximately 200,000 East Asians who were recruited from 66 hospitals at 12 medical institutions between 2003-2018. In a BBJ study with a 2-sample MR method, alcohol consumption was causally associated with HCC (odds ratio [OR], 1.57; 95% CI, 1.32 to 1.86) [22]. In the MEC study, which consisted of 215,000 males and females, including various races (e.g., Black, Native Hawaiian, Japanese American, Latino, or White), the amount of alcohol consumed was associated with colorectal cancer, and alcohol consumption also had a strong association with rectal cancer [23]. Compared to nondrinkers, those who drank more than 30 g/day had an OR for rectal cancer of 1.42 (95% CI, 1.61 to 1.75). This is consistent with our study results. In Korea, a case-control study of alcohol dehydrogenase 1B (*ADH1B*) (rs1229984) and aldehyde dehydrogenase 2 (*ALDH2*) (rs671), which are well-known genes that affect drinking behavior, and colorectal cancer was reported. The study concluded that the OR of colorectal cancer decreased in the group with a high level of allele A, which is related to a decrease in drinking [7].

This study has several limitations. First, the baseline age of the subjects in this study was around 41 years old, and even though the average follow-up period was 13.1 years, it still corresponds to a relatively young cohort with an average age of only the mid-50s. Therefore, the number of cancer types that occurred was not yet sufficient for a robust analysis. Second, among the 160,407 subjects who initially provided informed consent, only 129,324 subjects were included in the analysis, after the exclusion of other subjects who had missing alcohol intake data or who could not undergo genetic testing due to the lack of blood; this exclusion could have led to selection bias. In fact, the total cancer incidence was 817.9 in the group included in this study with complete information on drinking and smoking history variables, whereas it was as high as 1,078.3 in the group with missing drinking and smoking history variables. Third, there is a possibility that there was a measurement error in the amount of alcohol intake through the questionnaire itself. It is also possible that drinking-related genetic factors could not be found because the GWAS was conducted through drinking data that included measurement errors. However, in a Manhattan plot for the GWAS in this study, 2,240 SNPs with p-values < 5×10^-8^ were identified on chromosome 12, and the top SNP was rs75295329 (p=1.43×10^-306^) (Figure 1). In addition, it was confirmed that rs671, which is in a well-known drinking-related gene (*ALDH2*), is included in chromosome 12. However, in epidemiological studies, the amount of alcohol consumed according to the questionnaire may include measurement errors, and the amount of alcohol may be overestimated. Careful interpretation is needed because genetic factors related to the overestimated amount of drinking may be involved.

In conclusion, the results of this study suggest a limited causal relationship between GDAC and some liver and colorectal cancers. In particular, in the HBsAg-negative and elderly group, drinking causally increased the risk of liver cancer. For more reliable findings in the future, further studies should include a sufficient number of cancer types through long-term follow-up.

## Figures and Tables

**Figure f3-epih-45-e2023077:**
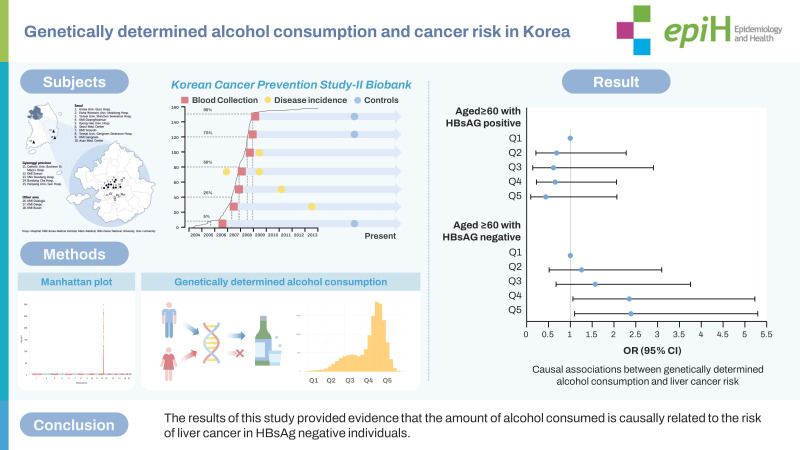


**Table 1.** General characteristics of the study population according to genetically determined alcohol consumption

Characteristics	Genetically determined alcohol consumption (g/day)					p-value
	Q1 (9.3±2.6)	Q2 (15.6±1.4)	Q3 (18.4±0.5)	Q4 (19.9±0.4)	Q5 (21.7±0.9)	
No. of cases/Total (n)	73/25,864	70/25,865	63/25,865	98/25,865	76/25,865	-
Age (yr)	42.0±10.5	41.5±10.4	41.2±10.3	41.1±10.3	41.4±10.3	<0.001
Body mass index (kg/m^2^)	23.4±3.1	23.4±3.2	23.6±3.2	23.7±3.2	23.6±3.2	<0.001
Amount of alcohol drinking (g/day)	7.9±16.7	14.5±23.7	19.0±27.0	19.2±27.1	19.4±27.3	<0.001
Sex (female)	38.5	37.7	39.1	38.1	37.7	0.423
Ever smokers (yes)	52.1	51.8	51.4	51.8	52.2	0.374
Exercise (yes)	42.0	41.7	41.7	41.5	41.4	0.659

Values are presented as mean±standard deviation or %.

**Table 2.** Causal associations between genetically determined alcohol consumption and cancer risk^1^

Variables		n	Genetically determined alcohol consumption, mean±SD (g/day)					p for trend
			Q1 (9.3±2.6)	Q2 (15.6±1.4)	Q3 (18.4±0.5)	Q4 (19.9±0.4)	Q5 (21.7±0.9)	
Cancer sites								
	All cancer	9,487	1.00 (reference)	1.06 (1.00, 1.13)	0.98 (0.92, 1.04)	1.03 (0.97, 1.10)	1.01 (0.95, 1.08)	0.992
	Esophagus cancer	53	1.00 (reference)	0.81 (0.39, 1.66)	0.26 (0.09, 0.81)	0.67 (0.30, 1.45)	0.56 (0.25, 1.26)	0.103
	Head and neck cancer^2^	140	1.00 (reference)	0.75 (0.46, 1.25)	0.65 (0.38, 1.11)	0.91 (0.56, 1.48)	0.76 (0.46, 1.26)	0.476
Colorectal		790	1.00 (reference)	1.39 (1.10, 1.74)	1.25 (0.98, 1.58)	1.50 (1.19, 1.89)	1.42 (1.13, 1.79)	0.003
	Colon	436	1.00 (reference)	1.33 (0.99, 1.80)	1.14 (0.83, 1.56)	1.36 (1.00, 1.85)	1.26 (0.93, 1.72)	0.165
	Rectum	372	1.00 (reference)	1.47 (1.04, 2.08)	1.38 (0.97, 1.96)	1.70 (1.21, 2.39)	1.60 (1.13, 2.25)	0.006
Liver		380	1.00 (reference)	1.05 (0.76, 1.46)	1.00 (0.72, 1.40)	1.49 (1.10, 2.02)	1.17 (0.85, 1.61)	0.048
Stomach		1,140	1.00 (reference)	1.02 (0.86, 1.23)	0.95 (0.79, 1.14)	1.00 (0.84, 1.20)	0.84 (0.70, 1.02)	0.108
Lung		669	1.00 (reference)	0.99 (0.78, 1.25)	1.05 (0.83, 1.32)	0.89 (0.70, 1.14)	0.96 (0.76, 1.22)	0.540
Thyroid		2,406	1.00 (reference)	0.99 (0.88, 1.13)	0.98 (0.87, 1.11)	0.91 (0.81, 1.04)	0.96 (0.85, 1.09)	0.279
Breast^3^		799	1.00 (reference)	1.04 (0.84, 1.28)	0.93 (0.75, 1.15)	0.90 (0.72, 1.12)	0.83 (0.67, 1.04)	0.062
Alcohol-related cancer^4^		2,061	1.00 (reference)	1.10 (0.96, 1.26)	0.98 (0.85, 1.12)	1.17 (1.02, 1.33)	1.07 (0.93, 1.22)	0.510

Values are presented as hazard ratio (95% confidence interval). SD, standard deviation.

1 Adjusted for age, sex and hepatitis B surface antigen (only liver cancer) using Cox proportional hazard model.

2 Included oral cavity cancer, pharynx cancer, larynx cancer.

3 Included only female cancer.

4 Included oral cavity cancer, pharynx cancer, larynx cancer, esophagus cancer, liver cancer, colon cancer, rectum cancer, and female breast cancer.

**Table 3.** Causal associations between genetically determined alcohol consumption and liver cancer risk^1^

HBsAg			n	Genetically determined alcohol consumption (g/day)					p for trend
				Q1	Q2	Q3	Q4	Q5	
Total (n)				25,864	25,865	25,865	25,865	25,864	-
PY				342,380	341,697	341,721	340,772	341,201	-
No. of case				73	70	63	98	76	-
Positive	All		196	1.00 (reference)	1.06 (0.70, 1.63)	0.87 (0.55, 1.38)	1.45 (0.97, 2.15)	0.94 (0.60, 1.46)	0.593
	Age (yr)	<50	128	1.00 (reference)	1.09 (0.60, 1.96)	0.97 (0.52, 1.78)	1.69 (0.98, 2.89)	1.31 (0.74, 2.30)	0.110
		≥50	82	1.00 (reference)	1.03 (0.56, 2.28)	0.75 (0.37, 1.52)	1.21 (0.67, 2.02)	0.49 (0.22, 1.10)	0.258
		<60	189	1.00 (reference)	1.14 (0.72, 1.80)	0.94 (0.58, 1.53)	1.63 (1.06, 2.51)	1.05 (0.65, 1.68)	0.320
		≥60	21	1.00 (reference)	0.69 (0.21, 2.28)	0.61 (0.13, 2.90)	0.66 (0.22, 2.06)	0.44 (0.09, 2.07)	0.285
	BMI (kg/m^2^)	<25	130	1.00 (reference)	1.30 (0.79, 2.19)	1.00 (0.58, 1.74)	1.32 (0.79, 2.20)	0.75 (0.41, 1.38)	0.511
		≥25	80	1.00 (reference)	0.65 (0.29, 1.46)	0.65 (0.28, 1.49)	1.61 (0.86, 2.82)	1.17 (0.59, 2.31)	0.100
Negative	All		170	1.00 (reference)	0.99 (0.59, 1.66)	1.12 (0.68, 1.96)	1.55 (0.97, 2.49)	1.42 (0.88, 2.28)	0.034
		<50	31	1.00 (reference)	1.01 (0.33, 3.14)	1.01 (0.33, 3.13)	1.53 (0.54, 4.29)	0.67 (0.19, 2.36)	0.901
		≥50	139	1.00 (reference)	0.98 (0.55, 1.75)	1.16 (0.66, 2.03)	1.53 (0.91, 2.61)	1.64 (0.98, 2.75)	0.015
	Age (yr)	<60	103	1.00 (reference)	0.88 (0.47, 1.64)	0.95 (0.51, 1.76)	1.21 (0.67, 2.17)	1.04 (0.57, 1.91)	0.556
		≥60	67	1.00 (reference)	1.26 (0.51, 3.09)	1.58 (0.67, 3.75)	2.35 (1.05, 5.23)	2.40 (1.09, 5.30)	0.008
	BMI (kg/m^2^)	<25	95	1.00 (reference)	0.78 (0.40, 1.55)	0.93 (0.48, 1.79)	1.29 (0.70, 2.38)	1.38 (0.76, 2.49)	0.115
		≥25	75	1.00 (reference)	1.34 (0.59, 3.01)	1.45 (0.65, 3.23)	1.98 (0.93, 5.23)	1.49 (0.68, 3.29)	0.181

Values are presented as hazard ratio (95% confidence interval). HBsAg, hepatitis B surface antigen; PY, person-year; BMI, body mass index.

1 Adjusted for age, and sex using Cox proportional hazard model.

## References

[1] Lewandowska AM, Rudzki M, Rudzki S, Lewandowski T, Laskowska B (2019). Environmental risk factors for cancer - review paper. Ann Agric Environ Med.

[2] Rumgay H, Murphy N, Ferrari P, Soerjomataram I (2021). Alcohol and cancer: epidemiology and biological mechanisms. Nutrients.

[3] Bagnardi V, Rota M, Botteri E, Tramacere I, Islami F, Fedirko V (2015). Alcohol consumption and site-specific cancer risk: a comprehensive dose-response meta-analysis. Br J Cancer.

[4] Bagnardi V, Rota M, Botteri E, Tramacere I, Islami F, Fedirko V (2013). Light alcohol drinking and cancer: a meta-analysis. Ann Oncol.

[5] Larsson SC, Carter P, Kar S, Vithayathil M, Mason AM, Michaëlsson K (2020). Smoking, alcohol consumption, and cancer: a mendelian randomisation study in UK Biobank and international genetic consortia participants. PLoS Med.

[6] Liu M, Jiang Y, Wedow R, Li Y, Brazel DM, Chen F (2019). Association studies of up to 1.2 million individuals yield new insights into the genetic etiology of tobacco and alcohol use. Nat Genet.

[7] Choi CK, Shin MH, Cho SH, Kim HY, Zheng W, Long J (2021). Association between ALDH2 and ADH1B polymorphisms and the risk for colorectal cancer in Koreans. Cancer Res Treat.

[8] Jee YH, Emberson J, Jung KJ, Lee SJ, Lee S, Back JH (2018). Cohort profile: the Korean Cancer Prevention Study-II (KCPS-II) Biobank. Int J Epidemiol.

[9] Cherukuri PF, Soe MM, Condon DE, Bartaria S, Meis K, Gu S (2022). Establishing analytical validity of BeadChip array genotype data by comparison to whole-genome sequence and standard benchmark datasets. BMC Med Genomics.

[10] Moon S, Kim YJ, Han S, Hwang MY, Shin DM, Park MY (2019). The Korea Biobank Array: design and identification of coding variants associated with blood biochemical traits. Sci Rep.

[11] Park S, Yoo HJ, Jee SH, Lee JH, Kim M (2020). Weighting approaches for a genetic risk score and an oxidative stress score for predicting the incidence of obesity. Diabetes Metab Res Rev.

[12] Kang MJ, Won YJ, Lee JJ, Jung KW, Kim HJ, Kong HJ (2022). Cancer statistics in Korea: incidence, mortality, survival, and prevalence in 2019. Cancer Res Treat.

[13] Jung KW, Kang MJ, Park EH, Yun EH, Kim HJ, Kong HJ (2023). Prediction of cancer incidence and mortality in Korea, 2023. Cancer Res Treat.

[14] Clarke TK, Adams MJ, Davies G, Howard DM, Hall LS, Padmanabhan S (2017). Genome-wide association study of alcohol consumption and genetic overlap with other health-related traits in UK Biobank (N=112117). Mol Psychiatry.

[15] Kang MJ, Jung KW, Bang SH, Choi SH, Park EH, Yun EH (2023). Cancer statistics in Korea: incidence, mortality, survival, and prevalence in 2020. Cancer Res Treat.

[16] Donato F, Tagger A, Gelatti U, Parrinello G, Boffetta P, Albertini A (2002). Alcohol and hepatocellular carcinoma: the effect of lifetime intake and hepatitis virus infections in men and women. Am J Epidemiol.

[17] Franceschi S, Montella M, Polesel J, La Vecchia C, Crispo A, Dal Maso L (2006). Hepatitis viruses, alcohol, and tobacco in the etiology of hepatocellular carcinoma in Italy. Cancer Epidemiol Biomarkers Prev.

[18] Jee SH, Ohrr H, Sull JW, Samet JM (2004). Cigarette smoking, alcohol drinking, hepatitis B, and risk for hepatocellular carcinoma in Korea. J Natl Cancer Inst.

[19] Song BG, Sinn DH, Kang W, Gwak GY, Paik YH, Choi MS (2022). Changes in the prevalence of hepatitis B and metabolic abnormalities among young men in Korea. Korean J Intern Med.

[20] Marengo A, Rosso C, Bugianesi E (2016). Liver cancer: connections with obesity, fatty liver, and cirrhosis. Annu Rev Med.

[21] Liu Z, Song C, Suo C, Fan H, Zhang T, Jin L (2022). Alcohol consumption and hepatocellular carcinoma: novel insights from a prospective cohort study and nonlinear Mendelian randomization analysis. BMC Med.

[22] Deng Y, Huang J, Wong MC (2022). Associations between six dietary habits and risk of hepatocellular carcinoma: a Mendelian randomization study. Hepatol Commun.

[23] Park SY, Wilkens LR, Setiawan VW, Monroe KR, Haiman CA, Le Marchand L (2019). Alcohol Intake and colorectal cancer risk in the multiethnic cohort study. Am J Epidemiol.

